# Biofilm Microbiome (Re)Growth Dynamics in Drinking Water Distribution Systems Are Impacted by Chlorine Concentration

**DOI:** 10.3389/fmicb.2018.02519

**Published:** 2018-10-23

**Authors:** Katherine E. Fish, Joby B. Boxall

**Affiliations:** ^1^Pennine Water Group, Department of Civil and Structural Engineering, The University of Sheffield, Sheffield, United Kingdom; ^2^NERC Biomolecular Analysis Facility, Department of Animal and Plant Sciences, The University of Sheffield, Sheffield, United Kingdom

**Keywords:** microbiomes, water quality, biofilms, disinfection, cleaning, bacteria, fungi, biological stability

## Abstract

Biofilms are the dominant form of microbial loading (and organic material) within drinking water distribution systems (DWDS), yet our understanding of DWDS microbiomes is focused on the more easily accessible bulk-water. Disinfectant residuals are commonly provided to manage planktonic microbial activity in DWDS to safeguard water quality and public health, yet the impacts on the biofilm microbiome are largely unknown. We report results from a full-scale DWDS facility used to develop biofilms naturally, under one of three chlorine concentrations: Low, Medium, or High. Increasing the chlorine concentration reduced the bacterial concentration within the biofilms but quantities of fungi were unaffected. The chlorine regime was influential in shaping the community structure and composition of both taxa. There were microbial members common to all biofilms but the abundance of these varied such that at the end of the Growth phase the communities from each regime were distinct. *Alpha-, Beta-*, and *Gamma-proteobacteria* were the most abundant bacterial classes; *Sordariomycetes, Leotiomycetes*, and *Microbotryomycetes* were the most abundant classes of fungi. Mechanical cleaning was shown to immediately reduce the bacterial and fungal concentrations, followed by a lag effect on the microbiome with continued decreases in quantity and ecological indices after cleaning. However, an established community remained, which recovered such that the microbial compositions at the end of the Re-growth and initial Growth phases were similar. Interestingly, the High-chlorine biofilms showed a significant elevation in bacterial concentrations at the end of the Re-growth (after cleaning) compared the initial Growth, unlike the other regimes. This suggests adaptation to a form a resilient biofilm with potentially equal or greater risks to water quality as the other regimes. Overall, this study provides critical insights into the interaction between chlorine and the microbiome of DWDS biofilms representative of real networks, implications are made for the operation and maintenance of DWDS disinfectant and cleaning strategies.

## Introduction

Drinking water distribution systems (DWDS) are designed and managed to (ideally) maintain the biostability of drinking water from treatment to tap, thereby safeguarding water quality and protecting public health. Biostability is generally agreed to refer to the maintenance of microbial water quality (Prest et al., 2016). As such, it is common practice to add a disinfection residual to final water to mitigate planktonic microbial (re)growth and contamination during distribution. However, the majority of the microbial biomass within DWDS is actually in biofilms (Flemming et al., 2002); mixed microbial taxa communities embedded within extracellular polymeric substances (EPS) which develop on the inner surfaces of the DWDS infrastructure. It is increasingly recognized that biofilms can degrade water quality by the processes that they mediate or their mobilization from the pipe wall into the water column, causing aesthetic quality failures and potentially presenting a public health risk if pathogens are released (Fish et al., 2016). The microbiota of biofilms has been established to be distinct from the planktonic microbiome (Henne et al., 2012; Douterelo et al., 2013; Roeselers et al., 2015), yet water quality standards and disinfection regimes only focus on planktonic microbial quality.

Chlorine is a widely used disinfection residual due to its low cost, ease of application and broad range of activation (Donnermair and Blatchley, 2003). Currently the World Health Organisation [WHO] (2003) recommends that biocide residuals (including chlorine) are used at concentrations no greater than 5 mgL^-1^, although disinfected drinking waters typically have concentrations between 0.2 and 1.0 mgL^-1^. However, chlorine efficacy is a function of more than concentration; pH, contact time, hydrodynamics, temperature, and chlorine demand all have an impact (de Beer et al., 1994). Much of our current knowledge regarding chlorination is based upon its action against planktonic cells and, at an operational level, disinfection application in water treatment – a bulk-water focused environment – in which the aforementioned parameters differ in comparison to the DWDS, which is a high surface-area-to-volume environment (Fish et al., 2016).

Despite the use of disinfection residuals, microorganisms remain in treated water, seeding the DWDS with a diverse community comprising bacteria, fungi, archaea, viruses, and amoeba (You et al., 2009; Thomas and Ashbolt, 2010; Lambertini et al., 2012; Douterelo et al., 2013; Potgieter et al., 2018). These microorganisms colonize the DWDS internal surfaces, or attach to existing biofilms, even if they are inactivated/injured (LeChevallier et al., 1996). The presence of a chlorine residual has been reported to be ineffective in preventing biofilm development (Chandy and Angles, 2001; Williams and Braun-Howland, 2003; White et al., 2011; Su et al., 2018) and may even promote biofilm formation as increasing chlorine concentrations within a model DWDS resulted in microorganisms favoring the biofilm state (Srinivasan et al., 2008). Disinfectant concentrations decrease in DWDS with increasing distance through the network (unless chlorine boosting is implemented) because the residual decays, which can also promote microbial activity. Residual chlorine may also indirectly support microbial growth by increasing assimilable organic carbon (AOC) concentrations due to organic carbon break down, which can also produce disinfectant by-products (DBPs) (Escobar and Randall, 2001).

Biofilm bound bacteria and fungi have been established to have increased resistance to residuals and tolerate greater disinfection concentrations than their planktonic counterparts, across various ecosystems (Costerton et al., 1995; Wingender et al., 1999; Hageskal et al., 2012, 19). The exact mechanisms behind this are still debated but there is a general consensus that the EPS provide physical protection in the form of a barrier (Chen and Stewart, 1996; Mah and O’Toole, 2001; Neu and Lawrence, 2009; Wang Z. et al., 2012; Xue et al., 2013). The resistance of biofilms to disinfection increases the chlorine demand of DWDS, leading to increased chlorine application, which can in itself impact water quality esthetics (taste and odor) as well as elevating operating costs (Chandy and Angles, 2001). Therefore, DWDS biofilms are of global concern as they present an unmonitored reservoir of microorganisms, with increased resistance to residuals, which can degrade water quality (biostability) and influence public health, both *in situ* (via the processes they mediate, e.g., oxidation/reduction, bio-corrosion causing leaching) and if they are detached into the water column (often a large scale, chronic impact on water quality where microorganisms, EPS and associated material is mobilized).

The impact of residual chlorine upon the ubiquitous (but often overlooked) DWDS biofilm microbiomes in full-scale systems is still to be explored. Of particular relevance is understanding how the effect of chlorine upon biofilms may change through a network as the residual decays and the concentration changes. Previous studies have predominantly investigated the influence of disinfectants upon the planktonic bacterial community (Gomez-Alvarez et al., 2012; Wang et al., 2014b; Li et al., 2018; Mao et al., 2018; Potgieter et al., 2018). A few studies have assessed biofilms in simulated distribution systems and reported the microbiome to be influenced by the type of disinfectant used (chlorine or chloramines) but the hydraulics and scale of these systems (Santo Domingo et al., 2003; Wang et al., 2014a) are not representative of operational DWDS. Additionally, bench-top based studies have highlighted the impacts and interactions between chlorine and biofilms cultured with specific pre-selected drinking water bacteria (Gomes et al., 2016; Lin et al., 2017). The trends observed in these studies suggest that changes in disinfection can impact the quantity of bacteria (Lin et al., 2017) and cause microbial community shifts with respect to selecting for (or against) specific bacterial groups (Santo Domingo et al., 2003) or affecting the relative abundance of bacterial or eukaryotic taxa but not impacting the specific taxa present (Wang et al., 2014a). Possibly, an increased chlorine residual concentration could select for microorganisms with increased resistance/tolerance to disinfection, potentially resulting in biofilms which are very difficult to eradicate and better able to shelter potential pathogens. Therefore, there is a need to determine the impact of disinfection concentration upon biofilms that are relevant to operational DWDS (i.e., natural mixed-species biofilms), while considering the complex abiotic and biotic interactions that occur within DWDS, which could influence disinfection and shape the microbiome.

It is important to recognize that current biofilm control strategies are an indirect consequence of other operational practices such as the use of chlorine residuals to manage planktonic microorganisms, or the mechanical cleaning of pipelines by using high flow rates to remove material from the pipe walls (Friedman et al., 2002; Husband and Boxall, 2011). As these approaches were not designed specifically for biofilm control their efficiency in managing biofilm development and persistence is uncertain and there are no guidelines for the protocols or frequency of such interventions. For this reason a critical next step in further understanding the biofilm-chlorine dynamics in DWDS is to explore the impact of “cleaning” on DWDS biofilms.

The primary aim of this study was to determine the impact of chlorine concentration upon the DWDS biofilm microbiome, specifically to assess the effect of chlorine upon bacterial and fungal quantities, community structure and taxonomic composition. A secondary aim was to compare the initial development of the microbiome to the subsequent regrowth of the bacterial and fungal communities (again under different chlorine concentrations) following a mechanical cleaning intervention.

## Materials and Methods

### DWDS Test Facility and Biofilm Growth

**FIGURE 1:**
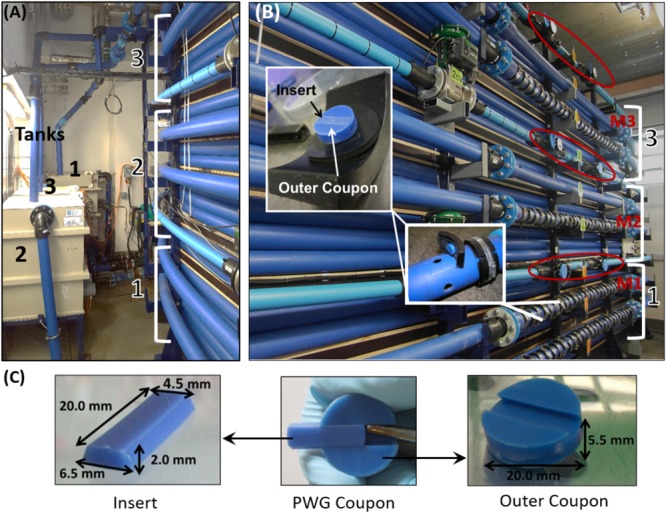
Drinking Water Distribution System Test Facility in which biofilms were developed. **(A)** The three independent tanks (0.49 m^3^) and high density polyethylene (HDPE) loops of length 200 m and internal diameter of 79.3 mm, **(B)** The sections of each loop into which PWG coupons were inserted; M1–M3 indicate the location of the online chlorine meters, red circles highlight the location of flow meters used to control the hydraulics, and **(C)** Details of the PWG coupon dimensions. Figures are adapted from Fish et al. (2015, 2018).

Biofilms were developed under different residual chlorine concentrations within a full-scale, temperature controlled DWDS test facility at The University of Sheffield, which has been described in detail previously (Fish et al., 2015, 2018). Briefly, three independent systems comprising a tank and pipe loop, along with online flow-, turbidity-, and chlorine-meters (Figure 1) were fed with water from the local DWDS (surface water source from peat upland catchment, treatment works applying chlorine residuals to finished water). For this study the facility was run at 16°C, selected as representative of United Kingdom summer water temperatures when microbial activity and water quality events peak. Prior to the start of these experiments the test facility was aggressively cleaned using hyper-chlorination and a high-flow rate flushing as set out in Fish et al. (2015).

Drinking water was pumped around the loops via variable speed pumps, following a double-peaked diurnal flow profile (peak flow of 0.54 ls^-1^ and low “night” flow of 0.23 ls^-1^), which is the common residential pattern of demand. Each system had a 24 h turnover as described in Fish et al. (2015) to preserve a baseline of water quality parameters such as nutrient supply and disinfection residual. Tapping points along each pipeline, as well as in the tanks, allowed for spot samples of water quality, alongside online measurements. Various water quality parameters were monitored thought the experiment to ensure that the only differences between the loops were related to chlorine concentration.

Each of the three loops was equipped with Pennine Water Group (PWG) coupons (Deines et al., 2010), which facilitated biofilm sampling (Figure 1C). Biofilms were developed naturally (*i.e.*, no inoculum was used to seed the facility) over a 28-day Growth phase, which is indicative of initial colonization of a replaced or relined pipe. Subsequently, a mechanical cleaning intervention was applied to each of the three loops prior to a further 28-day period of Re-growth to assess the impact of the cleaning in combination with chlorine and determine the biofilm behavior of a recently cleaned pipeline, which has not been addressed before. The 28-day time frame for Growth and Re-growth was selected as a comprise between replication/sampling intensity and experiment longevity to make the best use of the coupons available (per loop) for biofilm sampling and microbial community analysis.

### Chlorine Regimes and Water Quality

Biofilms were developed under one of three different chlorine residual concentrations, referred to as Low-, Medium-, or High-chlorine. The Medium-chlorine regime was run as the control, the chlorine concentration of this condition was determined primarily by the incoming water (in addition to the trickle turnover and chlorine demand of the system), and as such experienced a natural variation in residual concentration. Chlorine concentration was either boosted (for the High-chlorine regime) or reduced (for the Low-chlorine regime) using a 1:15 (v/v) dilution of 12% sodium hypochlorite or 1% solution of sodium ascorbate (Vit-D Chlor, United States), respectively. Both dosing solutions were kept in the dark, changed every 3 days and were added into the tank of the appropriate loop at the point of the turnover feed, via a peristaltic pump (Watson and Marlow 505).

On average, the free chlorine concentration within the Medium-chlorine regime was 0.45 mgL^-1^ (±0.05) in the Growth phase and 0.35 mgL^-1^ (±0.05) in the Re-growth phase. In the High-chlorine regime the free chlorine concentration was boosted to an average of 0.80 mgL^-1^ (±0.16) in the Growth phase and 0.82 mgL^-1^ (±0.05) in the Re-growth phase. Low-chlorine had an average of 0.05 mgL^-1^ (±0.06) during the Growth phase and 0.03 mgL^-1^ (±0.05) over the Re-growth phase.

The three regimes were run co-currently (one per loop) to ensure that all the loops had the same water quality (and variation therein) other than chlorine concentration. Water quality was measured via weekly spot samples (*n* = 3). Across the Growth and Re-growth phases the averages of the parameters monitored were: water temperature of 13.15°C (±0.70), pH of 7.89 (±0.19), turbidity of 0.07 NTU (±0.04), an ORP of 571 (±40.25), and metal concentrations of 3.68 μg L^-1^ Mn (±0.20) and 19.02 μg L^-1^ Fe (±3.51). All water quality parameters complied with United Kingdom Drinking Water Inspectorate (DWI) regulatory standards.

### Cleaning Intervention and Re-growth

After the Growth phase, each of the three systems were cleaned by flushing the pipelines with an increasing flow rate (and hence shear stress) of water. This is a typical mechanical cleaning approach utilized globally by water utilities to remove material (chemical and biological) from within the DWDS to reduce aesthetical impacts on water quality (discoloration, taste, odor) and decrease the chlorine demand of the system, in order to better prevent microbial regrowth and improve biostability (Friedman et al., 2002; Husband and Boxall, 2011; Cook et al., 2015). Each system was flushed (*i.e.*, cleaned) independently by applying four increasing flow rates (0.74, 3.58, 5.10, and 6.29 ls^-1^) and hence increasing shear stresses (0.09, 1.12, 3.42, and 5.65 Pa) for a duration of five turnovers at each step. During the flushing any dosing (to boost or reduce chlorine) was stopped. Due to the time taken to conduct the flushing, the three systems were flushed sequentially within a 24 h period; therefore two systems experienced an extra 12–16 h growth with some samples taken in the early morning of Day 29 – these end of Growth phase samples will be collectively referred to as Day 28.

Immediately after the cleaning, each system was filled with fresh water, dosing was restarted and the systems were set running again for a further 28 days of development referred to as the Re-growth. Because of the sequential nature of the flushing, the start of the Re-growth phase was staggered between the three systems. Any samples taken from the Re-growth phase are denoted with “R-.”

### Biofilm Sampling

Biofilm samples (*n* = 5) were collected from each chlorine condition at three time points during the Growth and Re-growth phases by removing PWG coupons at Day 0 (<90 min within the facility, used as a control) or R-Day 0 (<90 min into the Re-growth phase, after the cleaning), Day 14 or R-Day 14 and Day 28 or R-Day 28. Due to the staggered start of the Re-growth, the mid-point of this phase was R-Day 13 or R-Day 14 depending on the system, these will be collectively referred to as R-Day 14 for clarity. Biofilm sampling at the end of the Re-growth phase was staggered over a 24 h period so that all the final samples were taken at R-Day 28. All biofilm samples were taken without draining the loops to limit the impact of sampling and sterile coupons were used to replace those that were sampled.

### DNA Extraction

The PWG coupons sampled were separated into their two components (Figure 1C). Biofilm was removed from the outer coupons by standardized brushing into 30 mL sterile PBS, the biofilm suspensions were concentrated by filtering through a 47 mm diameter, 0.22 μm pore nitrocellulose membrane (Millipore, United States), as described in Fish et al. (2015). Sterile coupons (*n* = 3) were used as negative controls for the biofilm suspension stage. All samples (*n* = 90) and controls were stored at -80°C prior to molecular analysis. DNA was extracted from all the samples and negative controls using the proteinase K chemical lysis method, with CTAB (hexadecyltmethyl ammoniumbromide) incubation (Zhou et al., 1996; Fish et al., 2015).

### Quantitative PCR (qPCR) Analysis for Enumerating Bacteria and Fungi

Total bacteria and fungi within the biofilms were enumerated by quantifying the copies of the 16S rRNA gene and ITS region using a StepOne qPCR system (Applied Biosystems). Bacteria were amplified using the primers Eub338 (5′- ACTCCTACGGGAGGCAGCAG-3′) and Eub518 (5′- ATTACCGCGGCTGCTGG-3′) (Lane, 1991; Muyzer et al., 1993; Douterelo et al., 2018). For fungi the forward primer ITS1F (5′-TCCGTAGGTGAACCTGCGG-3′) and reverse primer 5.8S (5′- CGCTGCGTTCTTCATCG-3′) were used (Vilgalys and Hester, 1990; Gardes and Bruns, 1993; Douterelo et al., 2018). Each gene was quantified by using internal standard curves prepared from environmental samples from drinking water biofilms (*R^2^* ≥ 0.984), following the procedure set out in Douterelo et al. (2018). Internal calibration standards (ICS) were included to normalize/calibrate qPCR data to and make inter-plate comparisons more reliable.

Samples, standards, ICS, and no-template controls were amplified in triplicate according to the QuantiFast SYBR Green PCR kit (Qiagen, United Kingdom). Each 25 μL reaction contained: 12.5 μL QuantiFast SYBR Green PCR MasterMix, 9 μL nuclease free water (Ambion, Warrington, United Kingdom), 1.25 μL of each primer (10 μM) and 1 μL of DNA template (or nuclease free water for the controls). The PCR cycling conditions were 95°C for 5 min, then 35 cycles of 95°C for 10 s and 60°C for 30 s. The number of gene copies was determined using the StepOne software. Due to the number of samples it was not possible to run all the samples on the same qPCR plate. Therefore, sets of samples between which we wanted to compare were run on the same plate, with some samples being run on multiple plates to allow for comparison with other sample combinations.

### Illumina Sequencing

Extracted DNA was sequenced at the NBAF facility at The University of Sheffield, using the Illumina MiSeq platform. The bacterial 16S rRNA gene was amplified using the forward primer 63F (5′- CAGGCCTAACACATGCAAGTC-3′) and reverse primer 518R (5′- CGTATTACCGCGGCTGCTCG-3′) (Girvan et al., 2003). Fungal ITS regions were amplified using the primer pair ITS1F (5′- CTTGGTCATTTAGAGGAAGTAA-3′) and ITS4 (5′- TCCTCCGCTTATTGATATGC-3′) (Hageskal et al., 2006). Bacterial and fungal primer sets were integrated with MiSeq barcodes. PCRs were carried out on a Simpliamp Thermocycler (Fisher Scientific, United Kingdom), according to the protocols in Fish et al. (2015). After amplification samples were tagged with unique ID sequences by running a short multi-plex PCR. Samples were then purified via an AMPure XP bead clean-up and quality controlled prior to sequencing. Sequencing was performed on the Illumina MiSeq platform using the standard protocols. Raw MiSeq data have been uploaded to the NCBI Sequence Read Archive under accession number SRP153025.

### Data Analysis

Unless otherwise specified all plots and statistical tests were carried out using Rv3.5 (R Development Core Team, 2008). Significance was set at *p* < 0.05.

#### Bacterial and Fungal Quantification

Due to unsuccessful qPCR amplification two of the 180 samples analyzed were excluded from downstream analysis of the qPCR data. Therefore replication was *n* = 5 for all sample groups and both taxa apart from the fungi sampled from High-chlorine R-Day 0 and R-Day 28 where *n* = 4. If a quantity of “0” (mainly in the controls) was calculated from the qPCR analysis this was reported if the run met the default quality controls of the StepOne software. The technical triplicates of each sample were averaged to generate a single value per sample.

ANOVA analysis was applied to determine any statistically significant differences between chlorine regimes or time points. To ensure that comparisons were as robust as possible data were only plotted on the same graph or statistically compared if they were generated from the same qPCR run, although the use of ICS does render inter-plate comparison possible.

#### Sequence Processing

There were some differences in the level of replication and sample size between sample points (Supplementary Table S1) due to unsuccessful sequencing attempts, which predominantly occurred at Day 0 and R-Day 14 and was likely due to negative DNA extraction and amplification. All the successful paired-end reads were quality controlled using Trimmomatic (Bolger et al., 2014) to remove short sequences and noisy reads, prior to alignment using FLASH, with a 5% mismatch threshold (Magoc and Salzberg, 2011). Any reads >250-bp were excluded as they could not be aligned. Given the variable length of the fungal ITS region the excluded reads for this gene were retained and analyzed separately as “unaligned” reads. The aligned amplicon sequences were extracted using MOTHUR (Schloss et al., 2009). USEARCH (Edgar, 2010) was used to check for (and eliminate) chimeras and singletons, de-replicate sequences and cluster the sequences at 97% identity. USEARCH and Rv3.5 were used to generate matrices of the molecular Operational Taxonomic Units (mOTUs) and their relative abundance, or presence/absence within each sample. The unaligned-fungal reads were combined (using the reverse complement of read 2) and filtered to remove any sequences that were in the aligned-fungi dataset to ensure we did not duplicate reads. The filtered, unaligned-fungal reads were then de-replicated, checked for chimeric sequences and clustered as stated previously. The cleaned bacterial sequences were classified using the SILVA database (Quast et al., 2013) and the cleaned fungal sequences were classified using the UNITE database (Kõljalg et al., 2013), a 95% confidence interval was applied in both instances and taxa were assigned using the lowest-common-ancestor algorithm in MEGAN (Huson et al., 2016).

#### Community Analysis

Ecological index values for richness (Chao1), evenness (Simpson, inverted) and diversity (Shannon) within samples from each chlorine regime at each time point were calculated for bacteria and fungi (based on the relative abundance of mOTUs) using the R packages VEGAN and fossil (Dixon, 2009). ANOVA or *T*-tests were applied to compare the ecological indices between chlorine regimes and time points. Additionally, the number of total unique mOTUs was determined (based on the presence/absence data) for both taxa and across all replicates within a chlorine regime, at each time point and visualized on Venn-diagrams using the R package VennDiagram.

Bacterial or fungal community resemblance at the mOTU (based on Bray-Curtis similarities of square-root transformed relative abundance data) between chlorine regimes was visualized at each time point on a non-parametric multidimensional scaling (nMDS) generated using the software PRIMER-E v6^1^. Hierarchical clustering (based on group averages) was also conducted, the initial clusters from this analysis were overlaid on the nMDS plots. The stress values for the bacterial nMDS plots were ≤0.14. The fungal nMDS plot stress values were ≤0.11, apart from Day 14 (0.17). Analyses of similarity (ANOSIM) were used to determine statistically significant differences between sample groups. The outputs from ANOSIM are a *p*-value and a global-*R* value (referred to hereafter as “R”), which has a value of 0–1 where 1 indicates that communities were completely different.

For each sample group the average relative abundance of different taxonomic levels (phyla, class, and genus) was calculated across the replicates. This data was normalized by expressing the data as percentages of the total average abundance within a sample group and plotted as stacked bar charts. The ANOSIM test was applied to determine the effect of chlorine regime and Growth/Re-growth on bacterial or fungal communities at the different taxonomic levels. SIMPER analysis (similarity percentage; PRIMER-E v6^1^) was used to evaluate the similarity and dissimilarity between sample groups (expressed as %) and determine which bacterial or fungal phyla, class, or genus were primarily responsible (threshold of ≥75%) for the discrimination of sample clusters observed in the nMDS.

## Results

### Biofilm Microbial Concentrations

#### Bacterial Quantification

**FIGURE 2:**
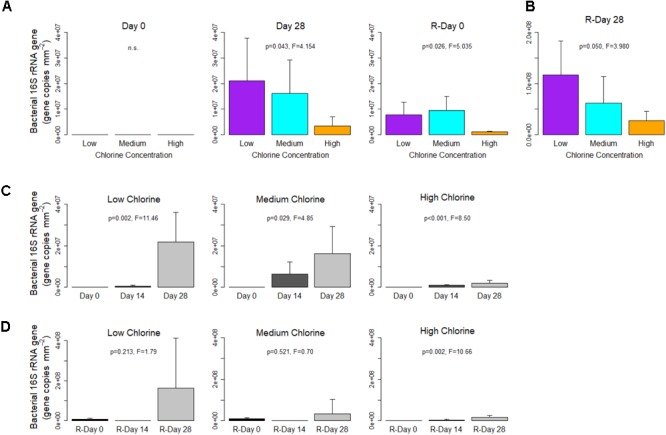
Bacterial gene concentrations within drinking water biofilms developed under different chlorine concentrations. **(A,B)** show the impact of chlorine concentration, note the different *y*-axis scale in **(B)**. For each regime, temporal changes are shown with respect to **(C)** Growth and **(D)** Re-growth phases. Data presented on a common plot were generated from the same qPCR run, *p*-values are from ANOVA analysis, n.s = no statistical significant difference, averages (*n* = 5) and standard deviation are presented.

At the end of the Growth and Re-growth phases biofilm concentrations of bacterial 16S rRNA gene copies were statistically significantly reduced as chlorine residual concentration increased (Figures 2A,B; note that data plotted together were derived from the same qPCR and can be directly compared). The impact of chlorine at Day 28 and R-Day 28 was driven by differences between the extremes of Low- and High-chlorine (*p* ≤ 0.042). Compared to Low-chlorine, which had an average of 2.18 × 10^7^ and 1.63 × 10^8^ 16S rRNA gene copies mm^-2^ at Day 28 and R-Day 28, High-chlorine concentrations reduced bacterial abundance in the biofilm by a magnitude. However, a substantial biofilm bacterial community still developed under the High-chlorine concentrations with (on average) 2.01 × 10^6^ and 1.75 × 10^7^ 16S rRNA gene copies mm^-2^ detected at Day 28 and R-Day 28, respectively. Although bacterial concentrations were greater at R-Day 28 than Day 28 for all the chlorine regimes (note the different *y*-axis scale Figure 2B), this increase was only statistically significant for the High-chlorine biofilms (Day 28 vs. R-Day 28; *T*-test, *p* = 0.021).

The temporal dynamics of bacterial concentrations are presented for each chlorine regime in Figures 2C,D, for the Growth and Re-growth, respectively. As expected, in all chlorine regimes, the number of bacterial gene copies increased from Day 0 (no genes detected in any regime) to Day 28 where there was a chlorine dose effect. During Growth the increasing bacterial concentration was linear where a chlorine residual was in place but an exponential pattern of growth was suggested in the Low-chlorine regime. In contrast, during the Re-growth, Low- and Medium-chlorine biofilms experienced a decrease in bacterial quantities between R-Day 0 and the mid-point (R-Day 14), before increasing again by R-Day 28, a trend which was less pronounced in the High–chlorine biofilms. Possibly this initial decrease in bacterial concentration was a delayed effect following the cleaning.

Data from R-Day 0 (Figure 2A) show that the cleaning intervention reduced the bacterial concentration compared to Day 28 but did not return the system to Day 0 conditions (Day 0 vs. R-Day 0; *T*-test, *p* ≤ 0.026). Low- and Medium-chlorine biofilms were more similar at R-Day 0 (*p* = 0.821) than Day 28, a result that indicates that the previous presence of a chlorine residual did not lead to a “cleaner” pipe (*i.e*., reduced biofilm presence) post-cleaning. Despite differences at R-Day 0, bacterial concentrations recovered so that R-Day 28 biofilms showed the same trends in bacterial concentration with respect to chlorine regime as were seen in Day 28 biofilms.

#### Fungal Quantification

**FIGURE 3:**
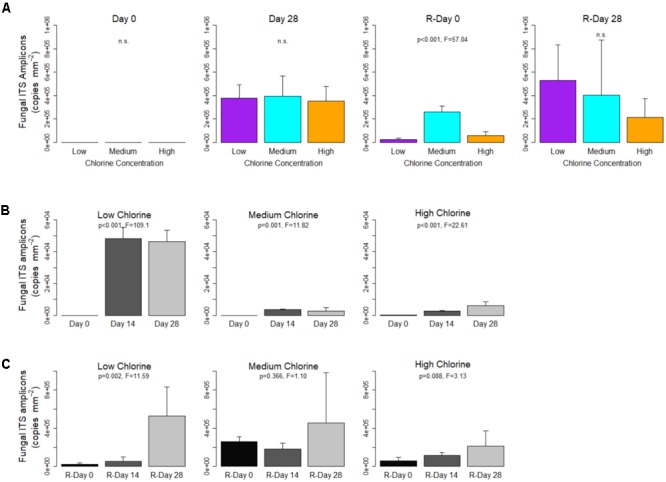
Fungal ITS region concentrations within drinking water biofilms developed under different chlorine concentrations. **(A)** The impact of chlorine concentration on gene concentration. For each regime, temporal changes are shown with respect to **(B)** Growth and **(C)** Re-growth phases. Data presented on a common plot were generated from the same qPCR run, *p*-values are from ANOVA analysis, n.s = no statistical significant difference, averages (*n* = 5 or *n* = 4 see section “Bacterial and Fungal Quantification”) and standard deviation are presented.

At each time point, for all chlorine regimes, the fungal ITS region was present at a concentration of at least one order of magnitude less than the bacterial 16S rRNA genes. Unlike the bacterial 16S rRNA genes, the concentration of fungal ITS amplicons did not differ significantly between chlorine regimes at the end of the Growth or Re-growth phases (Figure 3A). Although not significant, at R-Day 28 the average fungal concentration was shown to decrease with increasing chlorine concentration. Comparison of Day 28 and R-Day 28 biofilms showed that under Low-chlorine conditions there was a significant increase in fungal concentrations between the two time points (*p* = 0.023). Where a chlorine residual was present the fungal concentrations did not differ between Day 28 and R-Day 28 (*p* ≥ 0.075), therefore there was no dose effect of the chlorine in reducing fungal re-growth.

Figures 3B,C show the temporal dynamics of fungal gene concentration for each chlorine regime during Growth and Re-growth, respectively. Irrespective of chlorine residual concentration fungal ITS amplicon concentration increased between Day 0 (generally no genes detected) and Day 28. In the Low- and Medium-chlorine biofilms the greatest increase in fungal quantity was observed between Day 0 and Day 14, with little or no change in the final 2 weeks of growth, whereas in the High-chlorine regime fungal quantity continued to increase throughout the Growth phase (Figure 3B).

As was observed for bacteria, the cleaning intervention reduced the abundance of fungal gene concentration at R-Day 0 compared to Day 28 but did not return the system to Day 0 conditions (Day 0 vs. R-Day 0; *T*-test, *p* ≤ 0.045). Prior to cleaning there were no differences in fungal ITS amplicon concentration between the chlorine regimes, post-cleaning the Medium-chlorine biofilms contained a greater concentration (*p* < 0.001) than the Low- or High-chlorine regimes, which did not differ (*p* = 0.380). However, the recovery of the fungi was such that by R-Day 28 there were no significant differences between biofilms from the different chlorine regimes (Figure 3A).

### Community Structure and Resemblance (mOTU Level)

#### Bacterial Community

Illumina sequencing of the 16S rRNA gene yielded 35,077,377 reads post-trimming and quality control. The number of reads per sample ranged from 6343 to 1,968,671, with an average of 422,619. A total of 1,306 unique mOTUs were acquired (using a 97% similarity cut off) from the Growth and Re-growth samples. These mOTUs were analyzed to assess the bacterial community diversity and structure that developed under each chlorine regime.

**FIGURE 4:**
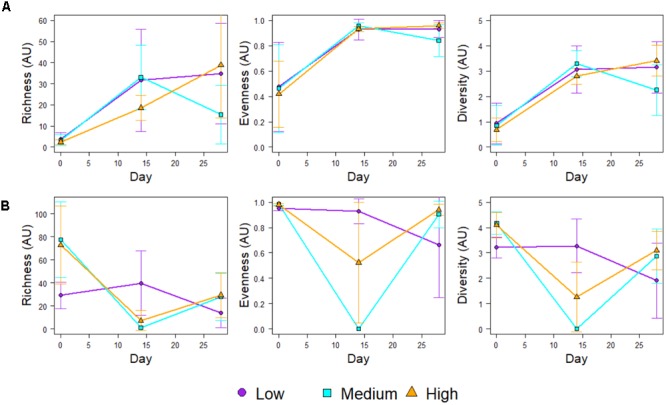
Ecological indices of the bacterial communities within biofilms developed under different chlorine concentrations during **(A)** Growth and **(B)** Regrowth, averages ± standard deviation are presented.

Figure 4 shows the community richness and diversity indices (based on the 16S rRNA mOTUs) for each chlorine regime, during the Growth and Re-growth phases. At Day-28 the greatest average richness, diversity and evenness were observed in the High-chlorine regime although these differences were not statistically significant. Indeed, during Growth there were no statistically significant differences in ecological indices between chlorine regimes. Conversely, chlorine regime has a statistically significant effect on the ecological indices of biofilms at R-Day 0 (*p* ≤ 0.026) and R-Day 14 (*p* ≤ 0.04). During the first 2 weeks of Re-growth the Medium- and High-chlorine bacterial communities decreased dramatically in their diversity, richness and evenness. In contrast Low-chlorine biofilms experienced little variation in their bacterial ecological indices. Between R-Day 14 and R-Day 28 the ecological indices of bacterial communities from the Medium- and High-chlorine increased, whilst the Low-chlorine indices decreased. This led to the indices of the three regimes converging at R-Day 28, although again the High-chlorine biofilms had the greatest average diversity, the differences seen were not statistically significant at the end of the Re-growth.

**FIGURE 5:**
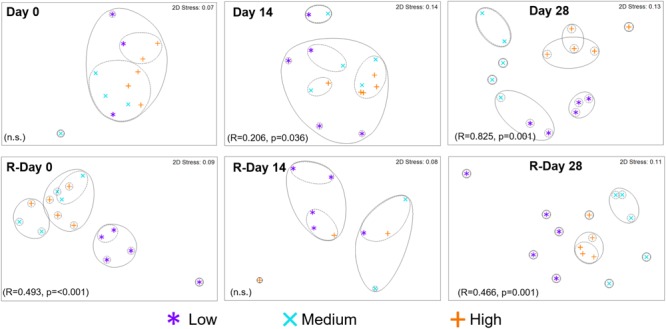
Resemblance between bacterial community structures from biofilms developed under three different chlorine conditions and sampled at the time points indicated. Growth and Re-growth data are presented, global-*R* and *p*-values are from ANOSIM analysis for chlorine effects. Full and dashed lines indicate clusters of at least 15 and 25% similarity, respectively, based on hierarchical clustering; n.s. = no statistically significant difference.

The resemblance between bacterial communities from each chlorine regime is shown in Figure 5. At the end of the Growth and Re-growth phases (Day 28 and R-Day 28) chlorine concentration had a statistically significant impact on bacterial communities such that each chlorine regime was distinct. High-chlorine bacterial communities were more similar to each other at both Day 28 and R-Day 28, than replicates from either the Medium- or Low-chlorine communities.

**FIGURE 6:**
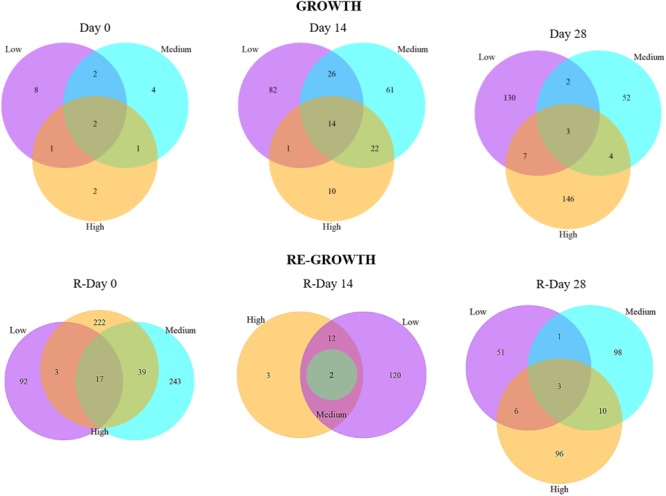
The number of shared and unique bacterial mOTUs within and between biofilms from different chlorine concentrations throughout the Growth and Re-growth phases. Venn diagrams are based on presence/absence mOTU data.

During Growth an increasing divergence between chlorine regimes was seen; at Day 0, there was no effect of chlorine regime (and only a few mOTUs were detected; Figure 6), by Day 14 there was a statistically significant difference between the Low- and High-chlorine regimes (*R* = 0.468, *p* = 0.016), which were clustering independently of each other, by Day 28 all three regimes were distinct (ANOSIM-pairwise tests: *R* ≥ 0.808, *p* = 0.008). Temporal variation in community resemblance during Re-growth was less linear. At R-Day 0 the Low-chlorine communities were distinct from the Medium- or High-chlorine regimes but at R-Day 14 there were no clear clusters based on chlorine concentration (at this sample point DNA was only amplified from five biofilm samples reflecting the decrease in bacterial concentration which was observed via qPCR and correlating with the reduction in ecological indices described previously). However, bacterial communities subsequently recovered such that R-Day 28 communities from each chlorine regime clustered independently. Comparing Day 28 and R-Day 28 bacterial communities revealed that under Low-chlorine conditions similar biofilm communities developed (*R* = 0.148, *p* = 0.111) but under Medium- or High-chlorine the communities were different (*R* ≥ 0.520, *p* = 0.008).

Following the cleaning intervention, the bacterial communities from the Medium- and High-chlorine regimes were similar (*R* = -0.008, *p* = 0.492) but the Low-chlorine remained distinct (Figure 5, R-Day 0; ANOSIM-pairwise, *R* ≥ 0.688, *p* = 0.008). Note that irrespective of chlorine regime the R-Day 0 and Day 0 communities were consistently significantly different (*R* ≥ 0.378, *p* ≤ 0.032). Similarly, ecological indices (Figure 4) did not differ between the Growth and Re-growth phases of any chlorine regime apart from statistically significant differences between Day 0 and R-Day 0, for which richness (*W* ≥ 20, *p* ≤ 0.020), evenness (*W* = 0, *p* ≤ 0.016) and diversity (*W* = 0, *p* ≤ 0.016) were significantly higher in the latter, for each regime.

Analysis of the presence/absence of mOTUs at each sample-point (Figure 6) also showed an increasing divergence between the chlorine regimes throughout Growth and Regrowth with the proportion of mOTUs found in all three regimes decreasing over time whilst the proportion of unique mOTUs increased. The mOTUs detected in all three regimes were assigned to the phyla Alphaproteobacteria *(Methylobacterium adhesium* and the genus *Rhiziobium sp.*) or Gammaproteobacteria (*Aeromonas sp.* and *Pseudomonas sp.*), with one mOTU unassigned. The Low- and Medium- microbiomes appeared to colonize quicker with a more rapid increase in the number of unique mOTUs compared to the High-chlorine regime. Additionally, at R-Day 28 the highest proportion of unique mOTUs were found in the Medium- and High-chlorine biofilms (37.0% and 36.2%, respectively). The mOTUs only found in the Medium- and High-chlorine biofilms were predominantly members of the phyla *Alphaproteobacteria* and *Gammaproteobacteria*, although *Betaproteobacteria* and *Actinobacteria* were also present.

The data also demonstrate that the differences between chlorine regimes are not due to different abundances of the same mOTUs, rather there are mOTUs unique to each chlorine regime, as well as a common “base” community the proportion of which decreases over the Growth and Re-growth phases.

#### Fungal Community

After trimming and quality control the number of ITS reads obtained was 5,384,033, the average number of reads per sample was 73,754, with a minimum of 4,661 and a maximum of 289,150 reads. Grouping mOTUs at a 97% similarity cut off led to the acquisition of a total of 149 unique mOTUs from the Growth and Re-growth phases, a magnitude lower than were obtained for the bacterial 16S rRNA gene.

**FIGURE 7:**
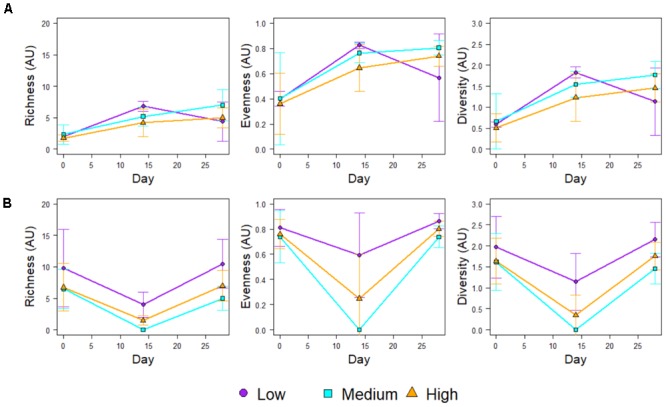
Ecological indices of the fungal communities within biofilms developed under different chlorine concentrations during **(A)** Growth and **(B)** Regrowth, averages ± standard deviations are presented.

Figure 7 shows the community richness and diversity indices for biofilms developed under the different chlorine concentrations, which were calculated using the relative abundance of the ITS mOTUs detected at each time point. Generally the richness, diversity and evenness of the fungal communities decreased with increasing chlorine concentration, although these differences were not statistically significant. The only biofilms for which this was not observed were the Day 28 biofilms in which the Low-chlorine condition selected for a less diverse fungal community with a lower evenness and reduced richness compared to the Medium- and High-chlorine biofilms. However, these differences were also not statistically significant.

Considering the temporal variation in richness, evenness and diversity (Figure 7) all three ecological indices doubled in the first 2 weeks of growth regardless of chlorine regime, continuing to increase steadily between Day 14 and Day 28 in the Medium- and High-chlorine biofilms but decreasing in the Low-chlorine biofilms, possibly due to competition. Fungal community richness (*W* = 0, *p* ≤ 0.033), evenness (*W* = 0, *p* ≤ 0.036) and diversity (*W* = 0, *p* ≤ 0.036) were statistically significantly higher at R-Day 0 than Day 0 highlighting that cleaning does not reset the fungal community structure to that of the primary colonization stage. The ecological indices then decreased in the first 2 weeks of Re-growth as shown in Figure 7B. Subsequently, the community structure regrew and returned to the same or higher diversity and evenness at R-Day 28 as was observed at Day 28. Although the R-Day 28 communities had greater diversity and richness with a more even structure than at Day 28 these differences were only statistically significant for the Low-chlorine biofilms (*W* = 1, *p* = 0.032).

**FIGURE 8:**
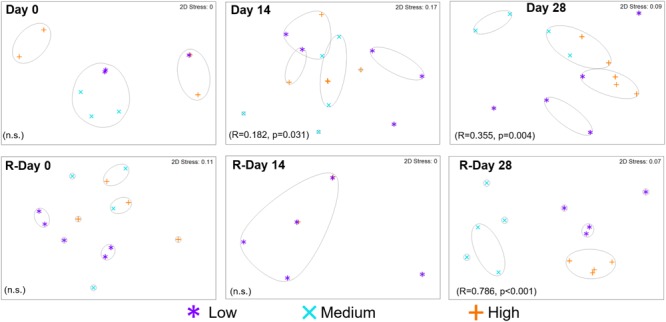
Resemblance between fungal community structures from biofilms developed under three different chlorine conditions and sampled at the time points indicated. Growth and Re-growth data are presented, global-*R* and *p*-values are from ANOSIM analysis for chlorine effects, gray lines indicate clusters of at least 30% similarity, based on group averages from hierarchical clustering; n.s. = no statistically significant difference.

**FIGURE 9:**
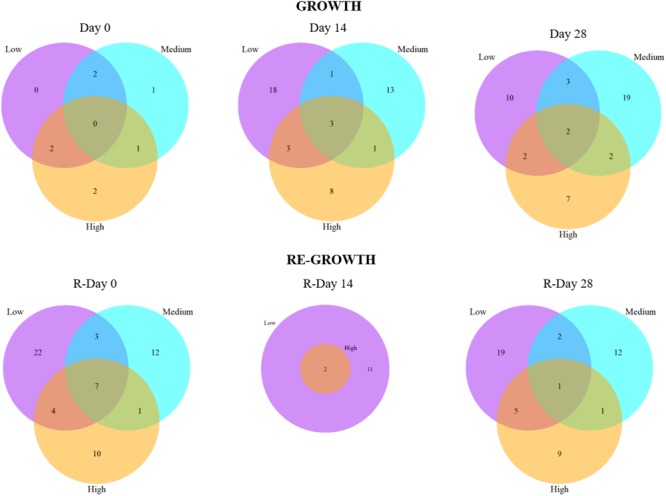
The number of shared and unique fungal mOTUs between biofilms from different chlorine concentrations throughout the **(A)** Growth and **(B)** Re-growth phases. Venn diagrams are based on presence/absence mOTU data.

Fungal communities did not differ between chlorine regimes at Day 0 (Figure 8) with only a few mOTUs being present (Figure 9). At Day 14 a chlorine effect was observed: Low- and High-chlorine fungal communities clustered independently and the Medium-chlorine fungal communities were similar to both. At Day 28 chlorine concentration had a statistically significant effect on fungal community as indicated in Figure 8, although Medium- and High-chlorine communities were more similar to each other than to the Low-chlorine as indicated by the hierarchical cluster analysis. High-chlorine biofilms had the least variation between replicates with respect to fungal community, as demonstrated by their tighter clustering (Figure 8).

At R-Day 0 and R-Day 14, there were no statistically significant chlorine effects on the fungal communities. Note that no fungal DNA was able to be extracted/amplified from the Medium-chlorine biofilms at R-Day 14, so comparisons are between Low- and High-chlorine only. Comparisons of the mOTUs detected at Day 0 and R-Day 0 highlighted statistically significant differences for the Low- and High-chlorine regimes (*R* ≥ 0.500, *p* ≤ 0.029) but not the Medium-chlorine (*R* = 0, *p* = 0.457), which could be influenced by the limited detection of fungal DNA at Day 0 (see Supplementary Table S1). At R-Day 28 fungal communities from each chlorine regime clustered independently and were significantly different. Regardless of chlorine regime, similar communities were present at Day 28 and R-Day 28 (*R* = 0.054, *p* = 0.109), indicative of similar communities developing at the end of Growth and Re-growth phases.

Figure 9 shows the presence/absence of mOTUs between chlorine regimes at each sampling point. At Day 0, the majority (62.5%) of the mOTUs detected were common to at least two chlorine regimes, this decreased dramatically at Day 14 (17.02%) and Day 28 (19.99%) as more unique mOTUs were colonizing biofilms within each chlorine regime. This was particularly obvious in the Low-chlorine condition which had no unique mOTUs at Day 0 and the most unique OTUs (18) at Day 14. During the Re-growth there was a decrease in the presence of mOTUs between R-Day 0 and R-Day 14, however, this is possibly influenced by the reduced sample size of the R-Day 14 time point for which no fungal DNA was found at detectable levels from any Medium-chlorine biofilm samples and only two High-chlorine samples had data (Supplementary Table S1). Day 28 biofilms from all three regimes contained two mOTUs which were assigned to the *Ascomycota* phyla and *Sordariomycetes* class, at R-Day 28 there was a single mOTU that was common in all three regimes (taxonomic information was not assigned to this mOTU). At the end of both the Growth and Re-growth phases the majority of mOTUs were unique to each chlorine regime, with High-chlorine having the lowest proportion of unique mOTUs and therefore a weaker influence over the fungal community composition than the other two regimes.

### Variation in Community Composition Based on Taxonomy

**FIGURE 10:**
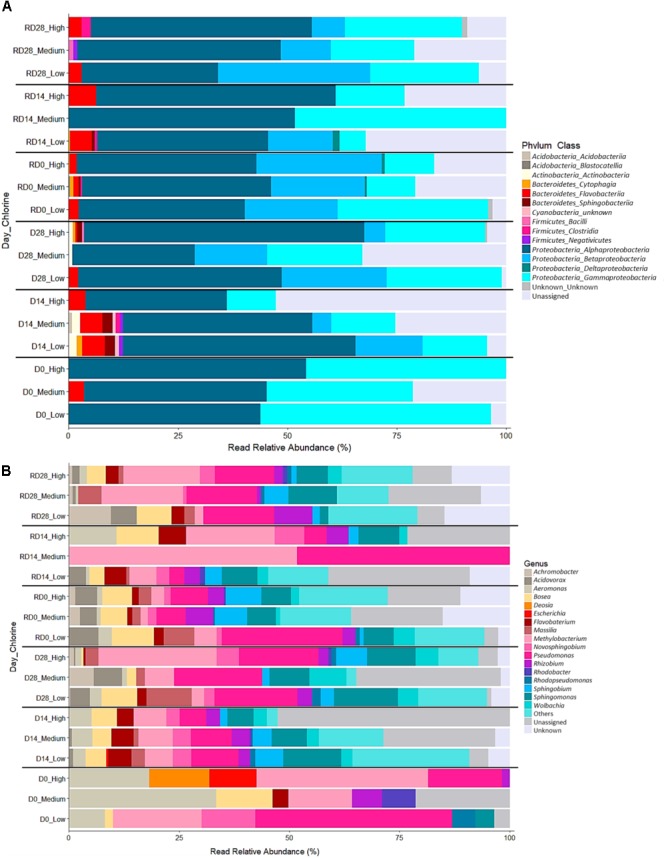
Relative abundance of bacterial classes **(A)** and genera **(B)** within biofilms from each chlorine regime, at each sample point. Classes in combination with the phyla they belong to (phylum_class) and genera are shown in the legend for each plot. “Unassigned” refers to sequences for which taxonomic information was unavailable, “Unknown” refers to sequences that were identified at a higher taxonomic level. “Others” in **(B)** includes 55 genera which were present at <5% relative abundance in any sample.

**FIGURE 11:**
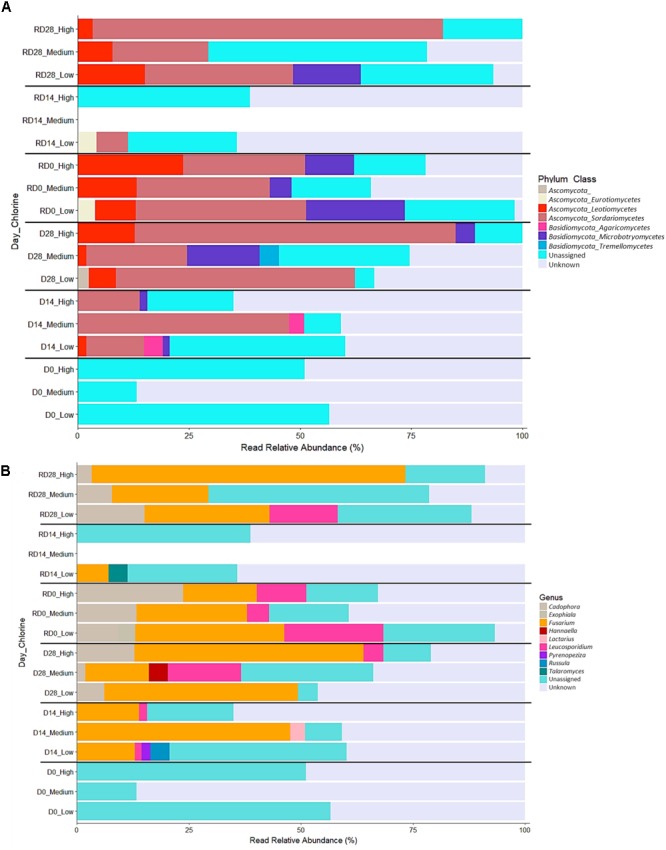
Relative abundance of fungal classes **(A)** and genera **(B)** within biofilms from each chlorine regime, at each sample point. Classes in combination with the phyla they belong to (phylum_class) and genera are shown in the legend for each plot. “Unassigned” refers to sequences for which taxonomic information was unavailable, “Unknown” refers to sequences that were identified at a higher taxonomic level.

The bacterial (Figure 10) and fungal (Figure 11) community composition was compared between biofilms from each chlorine regime, throughout the Growth/Re-growth with respect to phyla, class, and genus classification levels. SIMPER analysis was also applied to Day 28, R-Day 0 and R-Day 28 biofilms to identify the bacterial and fungal taxa (at phyla, class, and genus level) driving the differences between the chlorine regimes as observed in Figures 5, 8. These time points were selected based on the nMDS and ANOSIM analysis at the mOTU level showing differences between chlorine regimes at the end the Growth/Re-growth and R-Day 0 was included to determine any consistent impact on taxa of the mechanical cleaning.

#### Bacterial Community

##### Phyla and class level

Across all biofilm samples six bacterial phyla were detected. The majority of the bacterial mOTUs identified were members of the phylum *Proteobacteria* (85.30%), followed by *Bacteroidetes* (1.99%), and *Firmicutes* (0.84%). There were also a considerable proportion of mOTUs which could not be classified at the phylum level or below (11.18%). Most of the phyla were detected in more than one chlorine condition with the *Proteobacteria* present clearly dominating in all the biofilms. Although *Firmicutes* and *Actinobacteria* were consistently absent from the Day 28/R-Day 28 Low-chlorine biofilms.

Within the six phyla, 14 bacterial classes were detected, of which *Alphaproteobacteria, Betaproteobacteria*, and *Gammaproteobacteria* were predominant across all regimes and time points with respect to their relative abundance (Figure 9A). In combination, over 85% of the bacterial diversity in all the sampled biofilms was contributed by these three classes. At R-Day 0 *Flavobacteria* were also influential, being present at higher relative abundances in the Low-chlorine biofilms. The proportional abundance of *Alphaproteobacteria* was greatest in the High-chlorine biofilms at Day 28 and R-Day 28 but at R-Day 0 (after the cleaning intervention) the proportions were more similar between the chlorine regimes, although still slightly greater in the presence of chlorine compared to the Low-chlorine regime. Conversely, *Betaproteobacteria* decreased with increasing chlorine concentration within the Day 28/R-Day 28 biofilms and *Gammaproteobacteria* were less abundant in the presence of a chlorine residual across all the time points. Although High-chlorine biofilms retained a greater proportion of this class after cleaning than biofilms from the other two regimes the *Betaproteobacteria* population recovered more quickly in the absence of a chlorine regime. The bacterial classes discussed above explained 75% of the difference observed between chlorine regimes (SIMPER analysis), which was driven by variations in relative abundances. However, there were some classes which were not present in all chlorine regimes and explained a further 15% of the differences observed in Figure 4. Namely, *Sphingobacteriia* and *Clostridia*, which were unique to the High-chlorine biofilms, along with *Deltaproteobacteria, Cytophagia*, and *Actinobacteria*, which were absent from the Low-chlorine biofilms. ANOSIM analysis showed no significant differences between the bacterial communities at Day 28 and R-Day 28, for any of the chlorine regimes when assessed at the phyla or class level (pairwise ANOSIM, *R* ≤ 0.028, *p* ≥ 0.278).

##### Genus level

A total of 73 genera were found across all the biofilm samples, the most abundant of which were *Pseudomonas* (12.4% sequences), *Sphingomonas* (9.1% sequences), *Methylobacterium* (8.7% sequences), *Sphingobium* (5.5% sequences), *Bosea* (4.9% sequences), and *Massila* (4.5% sequences). *Pseudomonas* was common in all the biofilms but slightly more abundant in those from the Low-chlorine regime than the High-chlorine (Figure 9B). Of the genera detected 17, 25, and 21 explained 75% of the difference between the chlorine regimes at Day 28, R-Day 0 and R-Day 28, respectively. In each case 12, 18, and 11 of the genera driving the differences were present in biofilms from each regime at different abundances, the others were absent from at least one regime. Among the differentiating genera were *Massilia, Bosea*, and *Flavobacterium*, which were less abundant as chlorine concentration increased. Additionally, *Chromobacter, Rhizobium*, and *Acidovorax* were less abundant in the Medium- and High-chlorine biofilms. The abundance of *Methylobacterium* generally increased in the presence of a chlorine residual at the end of the Growth and Re-growth phases, although there was little difference between the Medium- and High-chlorine biofilms at R-Day 28. However, the *Methylobacterium* population within the Low-chlorine biofilms was more tolerant to the cleaning intervention because at R-Day 0 it was most abundant in the Low-chlorine regime. *Undibacterium* also contributed to the differences observed at R-Day 0 and was more abundant in High-chlorine regime than the Low-chlorine. Within each chlorine regime, the bacterial communities at Day 28 and R-Day 28 did not differ with respect to their genus composition (pairwise ANOSIM, *R* ≤ 0.156, *p* ≥ 0.135).

#### Fungal Community

##### Phyla and class level

Only two fungal phyla were identified from all the biofilms sampled throughout this study: *Ascomycota* (44.3% of mOTUs) and *Basidiomycota* (10.1% mOTUs), the remaining mOTUs could not be matched at the phylum level or below. Within these phyla, six fungal classes were detected across all the biofilms sampled, of which *Sordariomycetes* was generally the most abundant, followed by *Leotiomycetes* and *Microbotryomycetes* (Figure 10A). Unsurprisingly, the differences between biofilm communities at Day 28 and R-Day 28 (Figure 7) were predominantly due to variation in the abundance of these three classes (along with the unassigned mOTUs) under each of the three chlorine regimes as indicated by SIMPER analysis. Fungal communities did not differ significantly at R-Day 0 but there was variation in these three classes between the chlorine regimes. However, the trends were inconsistent. At Day 28 *Sordariomycetes* and *Leotiomycetes* were most abundant in High-chlorine biofilms and *Microbotryomycetes* was absent from Low-chlorine biofilms; at R-Day 28 *Sordariomycetes* was still most abundant in the High-chlorine biofilms but the abundance of *Leotiomycetes* was greatest in Low-chlorine and *Microbotryomycetes* were now only present in Low-chlorine biofilms. When the fungal communities at Day 28 and R-Day 28 were compared at the mOTU level a significant difference was detected in the Medium-chlorine regime. However, comparison at the phyla and class taxonomic levels found no differences in community composition between the two time points (pairwise ANOSIM, *R* ≤ 0.032, *p* ≥ 0.341).

##### Genus level

A total of nine fungal genera were identified in all the biofilms sampled, the most abundant of which were *Fusarium, Cadophora*, and *Leucosporidium* (Figure 10B). The differences between chlorine regimes were driven predominantly by these three genera in combination with the variation in the relative abundance of the unassigned sequences. While it is possible that these unassigned sequences represent fungi unique to DWDS, their substantial abundance indicates the need for more extensive fungal databases to improve taxonomic classification. The only consistent trend between fungal genera and chlorine concentration was that *Fusarium* was present in all the biofilms but most abundant in those from the High-chlorine regime. At Day 28 differences between the chlorine regimes were also driven by *Cadophora*, which was most abundant in the High-chlorine biofilms and *Leucosporidium* which was unique to the Medium- and High-chlorine biofilms. These trends were inversed in the R-Day 28 biofilms; the abundance of *Cadophora* reduced with increased chlorine concentration and *Leucosporidium* which was only detected in Low-chlorine biofilms. Despite these differences in certain genera, the composition of the Day 28 and R-Day 28 fungal communities did not differ statistically significantly (pairwise ANOSIM, *R* ≤ 0.383, *p* ≥ 0.05) for any of the chlorine regimes when assess at this taxonomic level.

## Discussion

### Chlorine Concentration Impacts on the Biofilm Microbiome

The current study presents novel insights into the impacts of chlorine concentration on a natural, mixed-taxa biofilm which has been developed under conditions relative to operational DWDS networks. At Day 28 (and R-Day 28) differences in biofilm microbial quantity and composition were evident between the chlorine regimes, indicative of a selective pressure likely being imposed by residual chlorine concentration because the hydraulics and other water quality parameters were consistent between the regimes and no differences were detected at Day 0.

It should be noted that the different chlorine regimes were all fed with the same microbial “inoculum” because they were supplied from the same inlet water source. Due to the characteristics of the local DWDS the incoming planktonic microbiome had already been exposed to chlorine residuals at concentrations most similar to the Medium-chlorine regime. It is likely that the incoming natural planktonic microbiome, which was the inoculum in this study, was already shaped by the chlorine-residual prior to entry into the test facility. Indeed, disinfectant regimes have been shown to have a selective pressure upon the planktonic bacterial communities within drinking water (Gomez-Alvarez et al., 2012; Wang H. et al., 2012; Wang et al., 2014b; Li et al., 2018; Potgieter et al., 2018). Yet the selective pressure of chlorine was so influential that it was still observable despite the inoculum being pre-conditioned to the Medium-chlorine concentration.

Increased chlorine residual concentrations decreased the quantity of bacterial genes within biofilms (a linear dose effect was observed between the Low-, Medium-, and High-chlorine regimes) but the concentration of fungal genes was unaffected by chlorine concentration (Figures 2, 3). These results suggest that fungi are more tolerant to chemical disinfection probably because they are morphologically more robust than bacteria. The thick, melanised cell wall of fungi conveys resistance to mechanical damage and limits the intrusion of biocides into the cell (Hageskal et al., 2012), whilst their ability to form spores also increases disinfection resistance (Sonigo et al., 2011). Indeed previous research has shown that fungi are tolerant to bacterial disinfection regimes (Zacheus et al., 2001; Hageskal et al., 2012) and may not be affected by water treatment processes because little difference exists between the raw and treated water fungal communities (Poitelon et al., 2009; Otterholt and Charnock, 2011; Sammon et al., 2011). Regardless of the chlorine regime, fungal genes were consistently less abundant than bacterial genes, often differing by an order of magnitude, which confirms previous reports that bacteria dominate the DWDS biofilm microbiome (Fish et al., 2015; Douterelo et al., 2018). It is important to note that while fungi may not dominate communities with respect to population size, fungi are larger than bacteria and so the fungal population may dominate with respect to biomass. Although not statistically significant, differences in the average concentration of the fungal ITS region began to emerge at R-Day 28 and mirrored the trends seen in the bacterial 16S rRNA gene in that there was a reduced average fungal quantity in the High-chlorine. It is possible that any effect of chlorine concentration upon fungal quantities within DWDS biofilms lags behind that of bacteria, possibly due to differences in generation time and cellular complexity. This is similar to the lag in fungal succession compared to bacterial succession which was reported by Douterelo et al. (2018). These results regarding bacterial and fungal quantification are unsurprising but this is the first time that the effect of different chlorine concentrations has been reported for DWDS biofilms developed under controlled, operational conditions.

Chlorine regime led to distinct microbiome community compositions but had little effect on community structures (ecological indices). Analysis suggested that the primary influence of varying chlorine concentration was to statistically significantly shift the abundance of a “core” bacterial or fungal biofilm community whilst selecting for taxa that were unique to the absence or presence of a chlorine residual was a secondary effect (Figures 5, 8, 10, 11). A shift in the abundance of the members of the “core” biofilm community was also reported by Wang et al. (2014a) when comparing chlorine and chloramines. The same bacterial and eukaryotic taxa were present in biofilms developed under the different disinfection agents but they were detected at varying relative abundances within biofilms, such that the communities were distinct from each other (Wang et al., 2014a). Irrespective of the chlorine regime, the dominant bacterial phyla was *Proteobacteria*, specifically the classes *Alpha-, Beta-*, and *Gamma-proteobacteria*, classes which have been found in many previous studies of DWDS planktonic and biofilm communities (Martiny et al., 2005; Yu et al., 2010; Henne et al., 2012; Liu et al., 2012; Douterelo et al., 2013, 2014, 2018). The abundance of certain bacterial classes varied between chlorine regime such that *Alphaproteobacteria* and *Methylobacterium* were more common at High-chlorine, *Betaproteobacteria* and *Gamma proteobacteria* were more abundant at Low-chlorine whereas *Pseudomonas* was found at similar abundances across the regimes. Potgieter et al. (2018) reported that the dominance of *Alpha-* or *Beta-proteobacteria* in the bulk water was dependent upon the type of disinfection but we found no consistent associations between dominance and chlorine concentration in the biofilms. Disinfection variation within model systems investigating biofilms has previously been shown to select for a specific bacterial group with ammonia oxidizing bacteria being promoted under chloramine compared to chlorine (Santo Domingo et al., 2003). Chlorination in particular has been reported to cause a shift from Gram-negative bacteria dominating in raw water toward Gram-positive bacteria dominating in treated water (Norton and LeChevallier, 1998). This may be due to the latter having a survival advantage due to differences in the cell structure (specifically the composition of the cell wall and peptidoglycan layer which is thicker in Gram-positive bacteria). Similarly to bacteria, the two fungal phyla (*Ascomycota* and *Basidiomycota*) detected herein have previously been reported in the few studies that have identified fungal taxa within DWDS biofilms (Zacheus et al., 2001; Heinrichs et al., 2013) and it was the variation in the abundance of these which drove the differences between the chlorine regimes. Interestingly the trends between chlorine concentration and fungal composition at Day 28 and R-Day 28 were inversed. For instance, *Leucosporidium* was initially detected in Medium- and High-chlorine biofilms at Day 28 but then was unique to the Low-chlorine biofilms at R-Day 28. This result suggests that chlorine is not having a consistent selective effect on the fungal community. Alternatively, the variation observed could be a stochastic effect based on the randomness of the abundance of species in the starting population and their transcriptional or epigenetic state, both of which would be dictated by previous environmental pressures. Alternatively, the inversed trends could be highlighting natural succession of fungal taxa in which the Low-chlorine fungal community lags behind the Medium- and High-chlorine communities because of the greater abundance and complexity of bacterial community in the Low-chlorine, which is outcompeting fungi for resources.

Analysis of both microbial taxa demonstrated a substantial proportion of sequences which were difficult to classify, this was particularly apparent for the fungal analysis. It is possible that this indicates the existence of novel bacteria and fungi adapted to the DWDS environment but clearly shows the need to expand the existing microbial databases at a fundamental level.

Chlorine regime did select for some unique taxa but these were generally less abundant than the “core” community and so had less of an impact upon the ecological indices which were similar between regimes, with the High-chlorine regime biofilms being slightly more diverse on average than the other regimes. However, there was less variation between replicates from the High-chlorine biofilms (with respect to bacterial and fungal communities) perhaps demonstrating a greater selective pressure being exerted by the higher chlorine residual concentration, leading to a more homogenous bacterial community. Nevertheless, the ecological indices did experience temporal variation, indicating differences in the rate and succession of growth between bacteria and fungi, as have been documented in detail previously (Douterelo et al., 2018) as well as highlighting a few impacts of chlorine concentration. Within the first 2 weeks of growth the founding community of both bacteria and fungi was established, as indicated by increased diversity and evenness. Subsequently, the fungal community was relatively stable whilst the bacterial communities continued to grow and diversify where a chlorine residual was present but decreased in the Low-chlorine regime. Although it has been suggested that increased disinfection reduces microbial diversity there is increasing evidence from pre-selected co-culture biofilms at the benchtop scale that multispecies biofilms exhibit greater resistance when compared to single species assemblages (Simões et al., 2010, 2016; Schwering et al., 2013). Our findings show, for the first time, that chlorine concentrations had similar impacts on community structure at the full-scale, for mixed-microbial taxa biofilms. With the currently available evidence it is not possible to determine the causation behind diversity promoting chlorine resistance. It could be that the higher chlorine selects for a more extensive EPS matrix which conveys greater protection to the microbial community, facilitating increased diversity in the microbiome or that a greater diversity in the microbiome is a pre-requisite for synthesizing the EPS to which chlorine protection is generally attributed (Neu and Lawrence, 2009). However, it is clear that under different chlorine concentrations biofilms could develop that present different risks to water quality (ranging from aesthetics such as discoloration, taste and odor, to endangering public health) if detached and could respond differently to cleaning interventions. Moreover, these results highlight the need to further understand the behavior of chlorine in inactivating organisms/biofilms. It is also critical that disinfection research is undertaken in more representative systems and with a more comprehensive view of the impact upon biofilms, incorporating wider taxa than simply bacteria and with consideration for the EPS and associated particles rather than just the microbiome.

### Combined Impacts of Cleaning Intervention and Chlorine on the Microbiome

The second aim of this study was to understand how biofilms (developed under different chlorine concentrations) were impacted by and re-grew after a mechanical cleaning intervention. This was assessed by comparing Day 28 and R-Day 28 biofilms. Regrowth of biofilms was anticipated to be accelerated compared to the Growth phase due to biofilm remaining post-cleaning, as has been reported previously (Fish et al., 2017; Gomes et al., 2018), therefore an established microbiome remained to recolonize the systems. However, High-chlorine was the only regime in which bacterial growth increased statistically significantly over the Re-growth, with a greater abundance of bacterial genes at R-Day 28 than Day 28 (although the High-chlorine regime still had lower bacterial concentrations compared to the other regimes at R-Day 28). The taxonomic composition of the biofilms at R-Day 28 was included in the detailed discussion and analysis above regarding the influence of chlorine upon growth. A key point to note is that for each chlorine regime the biofilm community composition and structure were similar between Day 28 and R-Day 28, when assessed at the various taxonomic levels (phyla, class, and genus), which demonstrates that cleaning does not alter the impact of chlorine concentration upon the microbiome.

The mechanical cleaning mobilized some of the microbiome but, unsurprisingly, did not “reset” the bacterial or fungal communities to Day 0 levels. The Low- and Medium-chlorine bacterial concentrations were similar at R-Day 0, as were the Low- and High-chlorine fungal concentrations. The fungal concentration within the Medium-chlorine biofilms was not as affected by the cleaning intervention as the other regimes, which could be a consequence of the medium chlorine concentration being the natural state for the incoming water, so the continually renewed inoculum is impacting the fungal development preferentially within this chlorine regime promoting the development of a more stable community. Overall, the previous presence of a chlorine residual did not improve the efficiency of the subsequent mechanical cleaning in reducing the bacterial or fungal communities. Gomes et al. (2018) compared the impact of sodium hypochlorite and mechanical cleaning on biofilms of *Acinectobacter calcoaceticus* and *Stenotrophomonas maltophilia* (isolated from drinking water) for which an extensive range of biofilm characteristics were assessed. The study concluded that, in general, pre-treatment with sodium hypochlorite (125 or 175 mg L^-1^) did not improve the impact of mechanical cleaning on biofilm removal (Gomes et al., 2018). Given that Gomes et al. (2018) evaluated chlorine concentrations that were much greater than those used in the current study, comparison of the two studies implies that even if water is distributed at the highest residual concentration recommended by World Health Organisation [WHO] (2003) (5 mg L^-1^), this would have a limited impact on restricting the development of the biofilm microbiome and no influence on the subsequent influence of mechanical cleaning.

The results from this study have highlighted a potential lag effect of the mechanical cleaning, which has not, to the authors knowledge, been reported previously. For each chlorine regime, both the bacterial and fungal communities experienced a general decrease in diversity, richness, and evenness (Figures 4, 7) at the mid-point of the Re-growth compared to R-Day 0 (the only exception to this was in the bacterial Low-chlorine biofilms). Similarly, at R-Day 14 bacterial gene concentration also decreased in comparison to R-Day 0 (Figure 2), although this was not as pronounced in the fungi there were fewer replicates at R-Day 14 (Supplementary Table S1) due to difficulties in obtaining sufficient fungal DNA from this time point. This Re-growth pattern contrasts those observed during the initial Growth phase for which the same parameters were increasing linearly or exponentially. We hypothesis that the mechanical cleaning removes some of the biofilm, potentially removing a substantial amount of the protective EPS which would impact the mechanical and chemical stability and resilience of the biofilms. The remaining microbiome would then be directly exposed to the chlorine residuals within the bulk-water which would be able to inactivate or kill the cells more easily, hence the dramatic decreases observed in the microbiome quantities and compositions. Synthesizing EPS requires energy so it likely that microorganisms would preferentially utilize the available resources (which could have been depleted by the removal of the EPS) to re-establish the matrix rather than investing in growth and replication. In so doing the biofilms would then be able to recover such that R-Day 28 and Day 28 biofilms were similar with respect to their microbiota. From the current study it is not possible to determine the time frame in which the delayed effect of cleaning was first occurring, this would require more frequent sampling in the first 14 days following the cleaning. Nevertheless, the trend described was a common finding across the various analyses of the microbiome and indicated that the cleaning was having the same impact in all cases. In order to explore this hypothesis it is essential to better understand the behavior of chlorine residuals within DWDS, their interactions with biofilm and the mechanisms by which the increased resistance of biofilms is conveyed. The results also demonstrate that while there is a need to understand the microbiome of DWDS biofilms and the impact of chlorine upon this, to get the most out of the data requires expanding such studies to include analysis of the physical structure (architecture and EPS) of biofilms.

### Practical Implications

The novel findings outlined in this study can be related to practical implications with regard to management of operational DWDS. Firstly, the presence of a High-chlorine concentration did not prevent biofilm formation and actually supported an increased amount of bacterial growth during the Re-growth, which suggests that limiting the use of a chlorine residual could be beneficial in certain networks. Operationally, the presence of a higher chlorine residual will affect the DWDS biofilm microbiome (quantity and species) influencing the amount of material that could be detached into the water-column and the potential risk it poses to biostability, at least in the short term. Secondly, the cleaning intervention was efficient in removing some of the microbiome and had similar effects across the three chlorine regimes. Furthermore, the microbiome was very similar between Day 28 and R-Day 28 across the chlorine regimes which implies that the mechanical cleaning conveys the same level of protection as aggressively cleaning a system (as was conducted prior to the start of the experiment), at least in the short term. The long term validity of this is not proven here as the microbiome was investigated over a relatively short time frame of 28 days. However, previous research regarding discoloration within DWDS, which is known to be strongly related to biofilm (Husband et al., 2016), suggests that regeneration of material within networks is linear with time (Husband et al., 2014; Cook et al., 2015). Hence the long term benefits from the simple mechanical cleaning and more aggressive cleaning may also be similar.

## Conclusion

Compared to low/no chlorine residual, an increased chlorine residual concentration has been shown to reduce the bacterial gene concentration within biofilms and also place a selective pressure upon the composition of the bacterial community colonizing the DWDS pipelines, at least in the short term. However, chlorine concentration did not influence the quantity of fungal genes within the DWDS biofilms, despite selecting for a distinct fungal community composition. The different behavior of fungi and bacteria demonstrates the need to consider and understand wider microbial taxa when exploring the DWDS microbiome, either biofilm or planktonic.

The effectiveness (based on the removal of biofilm microorganisms) of the mechanical cleaning was independent of the chlorine concentration. Following cleaning, a lagged decrease in the quantity and ecological indices of the bacteria and fungi of the microbiome were commonly observed. We suggest that this may be due to removing protective EPS which would impact the mechanical and chemical stability and resilience of the biofilms, which the biofilm then takes some time to recover from. There were generally no differences in the quantity or composition of the microbiomes which had developed by the end of the Re-growth phase, when compared to those at the end of the Growth phase. Together these factors imply that mechanical cleaning such as flushing is as efficient (at least in the short term) as aggressive cleaning (prolonged exposure to very high chlorine concentrations and extreme shear stress used here) in managing the biofilm microbiome within DWDS.

Interestingly the high-chlorine regime studied here was the only chlorine regime for which a significant increase in bacterial concentration was observed at the end of the Re-growth compared to the Growth, although the Re-growth concentrations were still the lowest of the three chlorine regimes. Hence, compared to the other regimes, in the long term the environmental pressures imposed by a higher chlorine residual may encourage the development of a highly resilient biofilm with as great, or greater, potential impacts on water quality.

## Author Contributions

KF and JB designed and planned the experiments, interpreted the results, and developed the discussion. KF conducted the experiments, carried out all the sample and data analysis, and wrote the manuscript. JB revised the manuscript.

## Conflict of Interest Statement

The authors declare that the research was conducted in the absence of any commercial or financial relationships that could be construed as a potential conflict of interest.

## Footnotes

1 http://www.primer-e.com/
