# Supplementary material for: Biofilm Microbiome (Re)Growth Dynamics in Drinking Water Distribution Systems Are Impacted by Chlorine Concentration
Source: Front Microbiol. 2018 Oct 23;9:2519. doi: 10.3389/fmicb.2018.02519 (PMC6232884; doi:10.3389/fmicb.2018.02519)
Supplement: Supplementary file 1 [file Data_Sheet_1.PDF]

Supplementary Material

Biofilm Microbiome (Re)Growth Dynamics In Drinking Water Distribution Systems Are Impacted By Chlorine Concentration

Katherine E. Fish* and Joby B. Boxall

*** Correspondence:** Corresponding Author: k.fish@sheffield.ac.uk

1 Supplementary Data

Table S 1 Details of replication for Illumina MiSeq analysis of bacterial and fungal communities from different sample groups, in all cases the number of samples (from n=5) is shown for which DNA was able to be extracted, amplified and sequenced successfully.

Sample Time Point	Chlorine Regime	Bacteria	Fungi
**Day 0**	Low	4*	3
	Medium	4	3
	High	5	4
**Day 14**	Low	5	5
	Medium	5	5
	High	5	5
**Day 28**	Low	5	5
	Medium	5	5*
	High	5	5
**R- Day 0**	Low	5	5
	Medium	5	4
	High	5	4
**R- Day 14**	Low	5	5
	Medium	2	0
	High	3	2
**R- Day 28**	Low	5	5
	Medium	5	5
	High	5	4

*One replicate later removed during data analysis as an outlier.

This replication is relevant to Figures 4-11 within the manuscript which are based upon Illumina sequencing data.
